# IL‐25 Disrupts the Nasal Epithelial Barrier in an Adapter Act1‐Dependent Manner

**DOI:** 10.1002/clt2.70165

**Published:** 2026-03-24

**Authors:** Baodan Zhang, Shenting Li, Qiqi Wang, Su Duan, Luo Zhang

**Affiliations:** ^1^ Department of Allergy Beijing TongRen Hospital Capital Medical University Beijing China; ^2^ Beijing Key Laboratory of Nasal Diseases Beijing Institute of Otolaryngology Beijing China; ^3^ Department of Otolaryngology Head and Neck Surgery Beijing TongRen Hospital Capital Medical University Beijing China; ^4^ Research Unit of Diagnosis and Treatment of Chronic Nasal Disease Chinese Academy of Medical Sciences Beijing China

To the editor:

Chronic rhinosinusitis (CRS) has become a significant public health issue that notably affects people’s health worldwide. The study further classifies CRS into localized type and diffuse type. Based on the differences in the intrinsic inflammatory response mechanisms of various types of lesions, diffuse CRS can be divided into eosinophilic CRS (eCRS) and non‐eosinophilic CRS (non‐eCRS), while the original diagnosis of chronic rhinosinusitis with nasal polyps (CRSwNP) is incorporated into diffuse CRS [1]. Each of the two aforementioned types of sinusitis can be further subdivided into two subtypes: with and without nasal polyps. Of these, the subtype with nasal polyps is strongly linked to epithelial barrier dysfunction and tissue hyperplasia driven by persistent chronic inflammation, and is also more refractory to curative therapy. In recent years, the concept of eosinophilic inflammation has been gradually evolving into the concept of type 2 inflammation [1]. Epithelial cells are a key regulatory hub in type 2 inflammatory networks: they are both targets of inflammatory injury and sensor‐secretory effectors. The barrier disruption by allergens, pathogens, or physicochemical insults prompts their rapid secretion of epithelium‐derived type 2 cytokines, which activate ILC2s and dendritic cells to initiate innate type 2 inflammatory responses. Meanwhile, IL‐13 from Th2 cells and ILC2s induces epithelial phenotypic remodeling, exacerbating tissue damage and forming a self‐sustaining inflammatory vicious cycle—initial epithelial barrier impairment triggers inflammatory signaling, which in turn further disrupts the barrier [2]. An intact and functional epithelial barrier characterized by tight junctions (TJs) plays a crucial defensive role in resisting the invasion of pathogens and pollutants. Zonula occludens‐1 (ZO‐1) is recognized as an essential core protein component of epithelial and endothelial tight junctions. Impairment in the structure and function of epithelial TJs is closely associated with the pathogenesis of chronic rhinosinusitis with nasal polyps.

IL‐25, as an inflammatory mediator in a variety of inflammatory responses, is closely associated with the occurrence of asthma, atopic dermatitis, and psoriasis. First identified in 2001, IL‐25 is mapped to chromosome 14q11 and belongs to the IL‐17 cytokine family. Target cells, which encompass diverse epithelial cell populations within interface tissues including the respiratory tract, gastrointestinal tract, genitourinary tract, skin and nasal mucosa (comprising ciliated epithelial cells, goblet cells, and basal epithelial cells), are the primary sites where IL‐25 initially binds to its cognate receptor interleukin‐17 receptor homolog 1 (IL‐17Rh1) to form a functional complex [3, 4]. The process by which IL‐25 induces diseases through disrupting the epithelial barrier has been extensively and thoroughly investigated in multiple organs, including the bronchi, skin, gastrointestinal tract, and thymus [3, 5]. However, its impact on the nasal mucosal epithelial barrier in CRSwNP has not yet been explored. As an essential factor for the activation of both NF‐κB and MAPK, Act1 is recruited to the receptor complex following the binding of IL‐25 to its receptor IL‐17Rh1 [6]. Accumulating evidence has demonstrated that the activated NF‐κB and MAPK pathways can further induce damage to the nasal mucosal epithelial barrier by regulating cellular processes such as apoptosis and stress responses [7, 8].

Thus, this study aims to investigate the expression of IL‐25 in nasal epithelial cells of CRSwNP and its role in the regulation of the nasal epithelial barrier. The subjects were divided into three groups using the JESREC scoring system [9]: the healthy control group (*n* = 15), the eCRSwNP patient group (*n* = 10), and the non‐eCRSwNP patient group (*n* = 25).

First, we performed immunohistochemistry on paraffin‐embedded samples from two CRSwNP patient subgroups and volunteers with healthy nasal mucosa, using mouse monoclonal anti‐human IL‐17Rh1 primary antibody and rabbit anti‐mouse IgG secondary antibody, to determine the presence of the IL‐17Rh1 receptor in CRSwNP nasal mucosa. We found that IL‐17Rh1 is expressed in the nasal mucosal epithelial cells of both eCRSwNP and non‐eCRSwNP patients (Figure 1A). A corresponding expression was also observed in the healthy controls, although the staining intensity relatively lower than that in the other two groups (Figure 1B,C).

**FIGURE 1:**
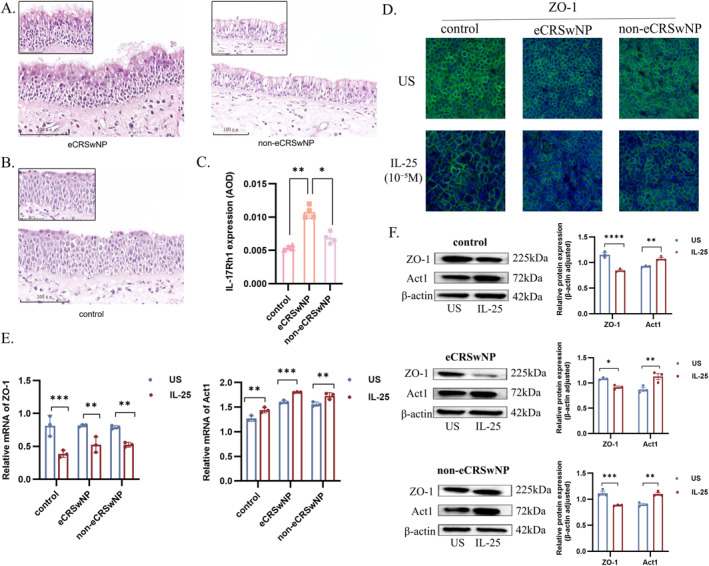
The effect of IL‐25 on the nasal mucosal epithelial barrier. (A) Immunohistochemical sections of nasal mucosa from patients with CRS. (B) Immunohistochemical sections of nasal mucosa from control group patients. (C) IL‐17Rh1 expression (AOD) across different groups. (D) Immunofluorescence staining of ZO‐1 in nasal mucosa‐derived cell cultures. (E) Relative mRNA levels of ZO‐1 and Act1. (F) Western blot analysis of ZO‐1 and Act1 proteins. *p < 0.05; **p < 0.01; ***p < 0.001; ****p < 0.0001. Data were acquired from at least 3 independent experiments and expressed as the mean ± standard deviation. Statistical analyses were performed using Student's *t*‐test, one‐ or two‐way analysis of variance (ANOVA). All statistical calculations were conducted with GraphPad Prism Version 9 Software (GraphPad Software, La Jolla, CA, USA), and statistical significance was defined as *p* < 0.05.

Primary human nasal epithelial cells (HNECs) were seeded into 24‐well plates containing PneumaCult‐Ex medium (Stemcell Technologies) and cultured at 37°C in a humidified incubator with 5% CO_2_. The recombinant human IL‐25 (IL‐17E) protein is a product of the PeproTech brand, distributed by Gibco (a brand under Thermo Fisher Scientific), with the catalog number 200‐24‐25UG (25 μg). Once the cells achieved full differentiation, they were stimulated with 10^−5^ mg/mL IL‐25 for 72 h. Immunofluorescence staining was subsequently performed on the cells in the 24‐well plates according to the protocols detailed below. Briefly, the cultured cells were fixed with a methanol‐acetone mixture (1:1, v/v), permeabilized with 0.3% Triton X‐100, and blocked with 5% non‐fat milk. The samples were incubated with a rabbit anti‐ZO‐1 polyclonal antibody (Invitrogen) and visualized under a confocal laser scanning microscope. Immunofluorescent staining of the TJ protein ZO‐1 was used to evaluate the integrity of the epithelial barrier. ZO‐1 in hNECs from the healthy control group appeared to be linearly localized along the cell junctions, while the other two groups showed discontinuous localization (Figure 1D). After stimulation with IL‐25, the organization and distribution of ZO‐1 in epithelial cells of each group were significantly disrupted compared with those in the untreated group.

Furthermore, quantitative real‐time polymerase chain reaction (qRT‐PCR) was performed to assess the transcriptional levels of TJ genes, with the qRT‐PCR kit procured from Takara. Concurrently, western blotting (WB) was employed to detect the expression of TJ‐associated proteins, using two primary antibodies: rabbit anti‐Act1 polyclonal antibody (working concentration: 1 μg/mL) and rabbit anti‐ZO‐1 polyclonal antibody (dilution ratio: 1:1000). Both antibodies were obtained from Invitrogen. We found that compared with the control group, the mRNA levels of the genes encoding ZO‐1 was significantly decreased in the IL‐25‐treated group (Figure 1E). Correspondingly, the evaluation of ZO‐1 protein expressed in cells from the healthy control group and the CRSwNP group showed that the expression of TJ was decreased in the IL‐25‐stimulated group, and the results of grayscale analysis of WB bands were statistically significant (Figure 1F).

Meanwhile, we found that compared with the control group, the relative mRNA and protein levels of Act1 were upregulated in the IL‐25‐treated group (Figure 1E,F). As an activator of NF‐κB, Act1 is recruited to the receptor after IL‐25 binds to its receptor, and participates in various downstream signaling pathways [6]. Activated NF‐κB orchestrates targeted inflammatory cascades in vivo and exerts regulatory control over cellular apoptosis and stress responses [7]. Furthermore, the MAPK signaling pathway, activated in an Act1‐dependent manner, induces mitochondrial apoptosis alongside oxidative stress and barrier dysfunction in placental cells [8].

In conclusion, IL‐25 has the potential to disrupt the nasal epithelial barrier in CRSwNP, and this effect may be associated with the activation of downstream NF‐κB and MAPK pathways. This finding enriches the pathogenesis of nasal polyps and identifies IL‐25 as a potential target for the diagnosis and treatment of CRSwNP.

## Author Contributions

**Baodan Zhang:** writing – original draft, investigation, formal analysis, visualization, software, validation. **Shenting Li:** investigation, data curation, formal analysis. **Qiqi Wang:** methodology, data curation, formal analysis. **Su Duan:** conceptualization, writing – review and editing, project administration, funding acquisition. **Luo Zhang:** conceptualization, resources, supervision.

## Funding

This study was supported by Capital Funds for Health Improvement and Research (2022‐1‐1091), Beijing Municipal Science & Technology Commission (Z211100002921057), CAMS Innovation Fund for Medical Sciences (2019‐I2M‐5‐022), the Program for the Changjiang Scholathe Program for the Changjiang Scholars and Innovative Research Teamrs and Innovative Research Team (IRT13082) and National Key Research and Development Program of China (2022YFC2504100).

## Conflicts of Interest

The authors declare no conflicts of interest.

## Data Availability

The data that support the findings of this study are available from the corresponding author upon reasonable request.

## References

[1] W. J. Fokkens , V. J. Lund , C. Hopkins , et al., “European Position Paper on Rhinosinusitis and Nasal Polyps 2020,” supplement, Rhinology 58, no. S29 (February 2020): 1–464, 10.4193/rhin20.600. 32077450

[2] Y. D. Gao , Z. J. Wang , I. Ogulur , et al., “The Evolution, Immunopathogenesis and Biomarkers of Type 2 Inflammation in Common Allergic Disorders,” Allergy 80, no. 7 (July 2025): 1848–1877, 10.1111/all.16620. 40586319

[3] J. Borowczyk , M. Shutova , N. C. Brembilla , and W. H. Boehncke , “IL‐25 (IL‐17E) in Epithelial Immunology and Pathophysiology,” Journal of Allergy and Clinical Immunology 148, no. 1 (July 2021): 40–52, 10.1016/j.jaci.2020.12.628. 33485651

[4] L. K. Ely , S. Fischer , and K. C. Garcia , “Structural Basis of Receptor Sharing by Interleukin 17 Cytokines,” Nature Immunology 10, no. 12 (December 2009): 1245–1251, 10.1038/ni.1813. 19838198 PMC2783927

[5] M. Hvid , C. Vestergaard , K. Kemp , G. B. Christensen , B. Deleuran , and M. Deleuran , “IL‐25 in Atopic Dermatitis: A Possible Link Between Inflammation and Skin Barrier Dysfunction?,” Journal of Investigative Dermatology 131, no. 1 (January 2011): 150–157, 10.1038/jid.2010.277. 20861853

[6] Y. Qian , C. Liu , J. Hartupee , et al., “The Adaptor Act1 Is Required for Interleukin 17‐Dependent Signaling Associated With Autoimmune and Inflammatory Disease,” Nature Immunology 8, no. 3 (March 2007): 247–256, 10.1038/ni1439. 17277779

[7] K. A. Kim , J. H. Jung , Y. S. Choi , and S. T. Kim , “Wogonin Inhibits Tight Junction Disruption via Suppression of Inflammatory Response and Phosphorylation of AKT/NF‐κB and ERK1/2 in Rhinovirus‐Infected Human Nasal Epithelial Cells,” Inflammation Research 71, no. 3 (March 2022): 357–368, 10.1007/s00011-022-01542-w. 35107605

[8] J. Bai , S. Deng , H. Fu , et al., “Chlorpyrifos Induces Placental Oxidative Stress and Barrier Dysfunction by Inducing Mitochondrial Apoptosis Through the ERK/MAPK Signaling Pathway: In Vitro and In Vivo Studies,” Science of the Total Environment 903 (December 2023): 166449, 10.1016/j.scitotenv.2023.166449. 37634732

[9] T. Tokunaga , M. Sakashita , T. Haruna , et al., “Novel Scoring System and Algorithm for Classifying Chronic Rhinosinusitis: The JESREC Study,” Allergy 70, no. 8 (August 2015): 995–1003, 10.1111/all.12644. 25945591 PMC5032997

